# Through the Looking Glass: Eye Tracking as a Cognitive Benchmark for Language-Model Semantics and Observer-Specific Semantic Alignment

**DOI:** 10.3390/jemr19050112

**Published:** 2026-10-02

**Authors:** Boris B. Velichkovsky, Ivan G. Mukhin, Indira R. Galeeva, Pavel Y. Sereda-Kalinin, Ivan A. Basyul, Yulia V. Bessonova

**Affiliations:** Institute of Psychology, Russian Academy of Sciences, 129366 Moscow, Russiaindigaleeva@gmail.com (I.R.G.); spyxp98@gmail.com (P.Y.S.-K.); basjulia@ipran.ru (I.A.B.);

**Keywords:** eye tracking, large language models, word embeddings, computational semantics, cognitive plausibility, semantic relatedness, surprisal, scanpaths, individual differences, representational similarity analysis

## Abstract

Assessing semantic representations learned by language models remains challenging because conventional intrinsic benchmarks capture limited aspects of meaning and often correlate weakly with downstream performance. Eye tracking offers complementary, cognitively grounded evidence: gaze measures recorded during reading and scene viewing index online processing and attentional selection, although they should not be treated as readouts of semantic representations. This narrative review and methodological framework synthesizes research linking distributional and contextual language representations to eye-movement behavior, focusing on semantic relatedness, predictability, surprisal, model scale, layer-dependent alignment, and model–human correspondence. Evidence from naturalistic reading corpora shows that embedding-derived relatedness and language-model surprisal explain variance in fixation measures, while recent work demonstrates that cognitive alignment depends on model size, layer, tokenization, task, and measurement regime. We propose extending this approach from population-level model evaluation to observer-specific gaze-derived relational structure. For visual scenes, gaze transitions can be represented as candidate choices and relational graphs and compared with multiple semantic-relation matrices derived from language or vision–language models. Because gaze transitions are influenced by spatial distance, object size, salience, task demands, context, and preprocessing decisions, these factors must be controlled and, where causal isolation is intended, manipulated independently of semantic relations. We propose combining candidate-normalized hierarchical choice models, residualized gaze relations, representational similarity analysis with reliability and uncertainty assessment, and required independent behavioral validation to characterize convergence and divergence between model semantic geometry and observer-specific relational organization.

## 1. Introduction: From Embedding Evaluation to Human Semantic Alignment

Vector representations of linguistic meaning have become foundational to natural language processing. Static embeddings such as Word2Vec and GloVe encode lexical regularities derived from distributional co-occurrence, whereas contextual models such as ELMo and BERT produce representations that vary with linguistic context. Contemporary large language models extend this trajectory by learning high-dimensional internal representations that support prediction, generation, reasoning, and multimodal processing. Yet the central evaluation problem remains: what does it mean for a semantic representation to be “good”?

Traditional intrinsic evaluations compare model similarity with human judgments or test vector analogies. These benchmarks are useful but narrow, sensitive to dataset construction, and not consistently predictive of downstream performance [1,2,3]. Extrinsic evaluation addresses practical usefulness by measuring performance on tasks such as named-entity recognition, question answering, or classification, but downstream accuracy is an indirect measure of representational quality because it reflects many components beyond semantics. The resulting gap has motivated cognitively grounded evaluation: model representations are evaluated according to how well they account for signals produced during human language processing [4].

Eye tracking is attractive in this context because it provides temporally precise, relatively unobtrusive measures of ongoing processing. During reading, first-fixation duration, gaze duration, total reading time, regressions, and skipping probability are shaped by lexical frequency, predictability, syntactic structure, and semantic integration [5,6,7,8]. During scene viewing, gaze is influenced by physical salience, spatial organization, goals, object identity, and semantic relations among scene elements [9,10,11]. These properties make eye movements informative, but also impose an important constraint: gaze is a behavioral consequence of multiple interacting processes, not a direct measurement of meaning itself.

This distinction is central to the present review. Rather than treating eye movements as a definitive “gold standard” for semantic representations, we treat them as a cognitively grounded benchmark that can reveal whether model-derived semantic variables explain behavior beyond established lexical, predictive, visual, and spatial factors. This framing also clarifies what model–gaze alignment can and cannot establish. A model that predicts eye movements well may capture cognitively relevant structure, but such success does not imply that its internal mechanisms are identical to human cognitive mechanisms.

The review has two linked aims. First, it synthesizes evidence on the relationship between distributional or contextual semantic representations and eye-movement behavior in reading, including recent work on gaze-informed NLP and model–human alignment. Second, it develops a more speculative but testable methodological framework: using gaze transitions among labeled objects in visual scenes to estimate observer-specific relational structure and compare that structure with the semantic geometry of language and vision–language models. The second aim is explicitly a framework for testing individual semantic alignment rather than a validated method for reconstructing an observer’s semantic representation.

Accordingly, the review asks five questions: (1) How have semantic representations been compared with eye-tracking data? (2) What do reading studies show about the separable roles of semantic relatedness and predictability? (3) What does alignment between model representations and gaze behavior establish about cognitive plausibility? (4) How can semantic, spatial, and visual influences on scene-viewing transitions be disentangled? (5) Under what conditions could gaze-derived relational structure be used to quantify individual human–model semantic alignment?

### Review Type, Scope, and Literature Identification

This article is a narrative review combined with a methodological framework rather than a systematic review. Literature identification was updated through 9 September 2026 using targeted searches of the ACL Anthology, PubMed, and publisher/index pages. Search combinations paired eye tracking, gaze, fixation, or scanpath with language model, transformer, word embedding, surprisal, semantic similarity/relatedness, cognitive alignment, semantic scene, area of interest (AOI), vision–language model, and representational similarity analysis. Selection was purposive: priority was given to peer-reviewed work that directly links eye-movement measures to computational language representations, gaze-informed NLP, cognitive model alignment, semantic scene/scanpath analysis, or methodological validity; foundational psycholinguistic and statistical sources were retained where needed to define constructs and analysis. Because the goal is conceptual synthesis rather than exhaustive retrieval, no PRISMA-style completeness claim is made. The review therefore distinguishes evidence that is established, evidence that is contingent on task/model/measurement choices, and hypotheses that require future validation.

## 2. What Is Being Evaluated? Semantic Similarity, Association, Predictability, and Representation

### 2.1. From Static Embeddings to Contextual and Large Language Models

Static distributional embeddings map each lexical type to a single vector. Word2Vec [12] and GloVe [13] encode lexical regularities accumulated across contexts, whereas contextual models such as ELMo [14] and BERT [15] assign a representation to a token in context. Large language models further complicate evaluation because ‘the semantic representation’ of a concept may depend on layer, tokenization, prompt, discourse context, model scale, and whether a dedicated embedding model or a generative hidden state is used. Consequently, comparisons with human behavior must specify what representation is extracted, from which layer or pooling procedure, and why that representation is theoretically appropriate.

At the sentence level, the mapping from symbolic linguistic input to a model-derived semantic representation involves several distinct stages. Text is first tokenized, contextual token representations are then generated by the selected language-model or transformer layer, and a sentence-level vector can subsequently be obtained through an explicitly specified pooling or sentence-embedding procedure. The resulting representation may then be used to derive semantic similarity or relatedness measures for comparison with behavioral data.

Cosine similarity remains a common tool for comparing vectors, but cosine similarity should not automatically be equated with human semantic similarity. Distributional proximity can reflect taxonomic overlap, thematic association, collocational regularity, contextual compatibility, or mixtures of these relations [16]. A review that asks whether gaze reflects ‘semantic distance’ must therefore define the construct more precisely than a single embedding metric and, when possible, compare competing relation models rather than treating one vector-space distance as ground truth.

### 2.2. Similarity Is Not the Same as Association

At least three relations should be separated. Taxonomic similarity concerns shared category structure (dog–cat; chair–sofa). Thematic or associative relatedness concerns concepts that participate in the same event or situation without being similar in kind (cup–coffee machine; dog–leash). Contextual coherence concerns how well a word or object fits a particular linguistic or visual context. These relations may correlate in natural data, but they are conceptually distinct and may guide gaze differently.

The distinction is especially important for scene viewing. A transition from knife to cutting board may be strongly motivated by functional association, whereas a transition from dog to cat may reflect categorical similarity. Treating both as instances of one undifferentiated “semantic similarity” measure risks obscuring the cognitive relation that actually guides attention. Future gaze-based semantic analyses should therefore compare multiple semantic models where possible, including taxonomic, thematic, contextual, and model-derived representations.

### 2.3. Predictability and Surprisal

A second distinction separates semantic relatedness from predictability. Surprisal is defined as the negative logarithm of the conditional probability of an upcoming word given its preceding context. Surprisal theory predicts increased processing difficulty for less expected words [17,18], and reading-time studies have repeatedly shown that language-model surprisal predicts fixation and reading-time measures [19,20]. Because distributional similarity and predictability are both shaped by co-occurrence, they are correlated; this makes it difficult to interpret an association between embedding similarity and gaze as a purely semantic effect.

Frank [21] showed that some apparent semantic-similarity effects on reading behavior can disappear once predictability is controlled, motivating strong caution about semantic interpretations. More recent work indicates that the relationship is not exhausted by a simple either/or account. Salicchi, Chersoni, and Lenci [22] reported separable contributions of surprisal and contextual BERT-derived relatedness, and Sun, Wang, and Lu [23] showed that interpretable contextual semantic-similarity measures can improve prediction of eye movements. At the same time, model scale is not monotonically related to human-like processing: surprisal from larger models can fit human reading times more poorly [24], training exposure can produce an intermediate optimum [25], and parameter size can relate differently to early and later eye-movement measures across languages [26]. Subword tokenization also matters because human measures are typically defined over words or regions that do not coincide with LM token boundaries; principled aggregation or marginalization is therefore required [27,28]. The appropriate conclusion is not that gaze ‘measures semantics,’ but that semantic relatedness and predictability can make partially distinguishable contributions whose magnitude depends on representation, layer, corpus, tokenization, metric, model scale, and statistical design.

## 3. Eye Movements During Reading as a Cognitive Benchmark

### 3.1. Why Reading Measures Are Informative

Reading consists of sequences of fixations and saccades rather than continuous visual uptake. Fixation durations and regressions reflect a mixture of lexical access, syntactic processing, prediction, integration, and reanalysis [5]. Semantic anomaly and reduced contextual fit can increase processing time [6,7]. This makes reading behavior a natural test bed for asking whether model-derived variables account for human processing difficulty.

The most commonly analyzed measures include first-fixation duration, gaze duration or first-pass reading time, total reading time, fixation count, regression probability, and go-past time. Earlier measures are often more strongly associated with lexical access and immediate processing, whereas later measures incorporate reanalysis and integration. These distinctions are probabilistic rather than absolute; no single eye-tracking metric uniquely identifies a cognitive process.

### 3.2. Public Reading Corpora

Several corpora have enabled model–gaze comparisons at scale. GECO contains naturalistic reading of Agatha Christie’s The Mysterious Affair at Styles in monolingual and bilingual conditions and provides multiple word-level eye-movement measures [29]. The Provo Corpus contains 55 short English passages read by 84 native speakers together with predictability norms [30]. The Russian Sentence Corpus provides benchmark eye-movement data for Russian sentence reading [31]. The Multilingual Eye-Movement Corpus (MECO) extends naturalistic passage reading across 13 languages, providing an important basis for cross-linguistic evaluation [32].

These resources differ in genre, linguistic diversity, participant population, text length, preprocessing, and task. Consequently, differences in model ranking across corpora should not automatically be interpreted as differences in semantic quality. Corpus-specific lexical distributions, language properties, participant strategies, and measurement reliability all affect the relationship between model predictors and gaze. Contemporary LM analyses add a further alignment problem: word-level fixation measures must be mapped consistently to subword-token predictions, and alternative alignment rules should be reported or tested for sensitivity [27,28].

### 3.3. Correlation-Based Evaluation

A common approach computes the semantic relation between a target word and its preceding context and correlates this value with eye-tracking measures. Salicchi, Lenci, and Chersoni [33], for example, compared static and contextual representations on naturalistic reading data by relating embedding-derived cosine similarity and language-model surprisal to gaze features. The expected direction for a facilitative relatedness effect is usually negative: stronger relatedness should be associated with shorter reading times. Yet this expectation is meaningful only when lexical frequency, word length, predictability, and other major covariates are considered [34].

Simple bivariate correlation is therefore best treated as descriptive rather than definitive. A stronger design uses regression or mixed-effects models to ask whether a semantic predictor explains variance beyond established controls and whether its effect generalizes across participants, items, and corpora.

### 3.4. Prediction-Based Cognitive Evaluation

CogniVal introduced a broader framework in which word representations are evaluated by their ability to predict cognitive signals from eye tracking, EEG, and fMRI [4]. Instead of reducing a vector to one similarity score, prediction-based evaluation can exploit the full representational structure. Hollenstein and colleagues showed that different embeddings vary in their ability to predict cognitive data, illustrating that cognitive evaluation can provide information not captured by conventional intrinsic benchmarks.

Prediction accuracy should nevertheless be interpreted carefully. A representation may predict gaze because it encodes word frequency, syntactic regularity, contextual expectation, or other correlates rather than semantic structure alone. Cognitive prediction is therefore strongest when accompanied by ablation analyses and baselines that isolate what information the representation contributes.

### 3.5. What Existing Comparisons Do—And Do Not—Show

Across the literature, both static and contextual representations show measurable relationships with eye-movement behavior. GloVe and BERT have sometimes produced comparatively strong associations in similarity-based analyses [33], while cognitive-prediction frameworks show that representations vary in their ability to predict eye tracking and other cognitive signals [4]. More recent studies broaden this picture: human dwell time has been associated most strongly with attention patterns in earlier transformer layers rather than with parameter count alone [35]; cognition-guided pretraining and gaze-informed attention can change downstream behavior [36,37]; and model scale or training exposure can either improve or degrade psychometric fit depending on the eye-movement measure and regime [24,25,26]. These findings support representation- and measurement-specific cognitive alignment, not a universal ranking of language models.

A more precise vocabulary is therefore preferable. Gaze alignment should be described as evidence of cognitive or behavioral correspondence under a specified task, representation, layer, tokenizer, corpus, and measurement regime. It does not establish that the highest-correlating model has the ‘best’ semantics in an unrestricted sense, nor that model attention is identical to human attention. Table 1 summarizes representative recent work illustrating how conclusions change with the comparison target and analysis design [38].

## 4. Model Attention and Human Gaze

Transformer attention and human gaze are frequently compared because both can be represented as distributions over elements in a sequence. This analogy is methodologically useful but cognitively hazardous if taken literally. Transformer attention is a learned computational operation within a model; eye movements are overt motor behavior constrained by visual acuity, oculomotor control, task goals, and cognition. Empirical comparisons reinforce this caution: attention–gaze correspondence varies across architectures and layers [35,41], and gaze-informed attention can improve some downstream tasks without implying mechanistic equivalence [36,37]. Similarity between model and gaze allocation may therefore reveal convergent structure or useful inductive bias, not identity of underlying mechanisms.

Dini et al. [42] examined whether transformer models encode information related to human reading behavior and whether intermediate fine-tuning on eye-tracking features changes model alignment. Their results indicate that gaze-related information is present in pretrained transformer representations and that eye-tracking fine-tuning can increase model–human alignment in the evaluated setup. Recent gaze-informed NLP similarly demonstrates that human or synthesized scanpaths can contribute useful signals [37,43,44]. These results expand cognitive evaluation from output correlations toward analyses of internal and task-adapted model structure, but the inference should remain one of representation-specific behavioral alignment rather than identity between model attention and human attention.

## 5. From Reading to Visual Scene Processing

### 5.1. Semantic Guidance in Scenes

Natural scene viewing provides a complementary domain because fixations are directed toward objects and regions rather than ordered words. Gaze selection in scenes is jointly influenced by bottom-up visual properties, spatial structure, task demands, prior knowledge, and semantic expectations. Hwang, Wang, and Pomplun [9] demonstrated semantic guidance of eye movements in real-world scenes, including a tendency for successive fixations to be influenced by semantic relations among scene objects. Visual-world studies likewise show that spoken language can attract gaze toward semantically related objects, demonstrating that semantic activation can affect overt visual attention [10].

These findings justify treating object-to-object transitions as potential carriers of semantic information, but not as pure semantic measurements. Nearby objects are easier to reach with short saccades; large or visually salient objects attract fixation; scene roles constrain plausible transitions; and tasks radically reshape scanpaths. Moreover, object–scene semantic consistency does not produce a single invariant attentional signature across paradigms [45,46]. Any attempt to infer semantic organization from scene viewing must therefore model these influences explicitly and, when the goal is to isolate a semantic effect, should prefer controlled or counterbalanced stimuli in which semantic relations and spatial arrangement are varied independently.

In this framework, an individual fixation is therefore treated as an observed attentional event associated with a word, object, or AOI rather than as a semantic event in itself. Semantic information is inferred from structured patterns of fixations and transitions only after visual, spatial, task-related, and oculomotor influences have been considered.

### 5.2. Scene-Viewing Datasets

The OSIE dataset contains eye-tracking data for 700 natural images together with object-level semantic annotations, making it well suited to analyses that connect fixations to labeled objects [47]. COCO-Search18 provides approximately 300,000 search fixations from 10 participants searching for 18 target-object categories in 6202 COCO images [48]. Its scale and object annotations make it valuable for modeling goal-directed scanpaths, although visual search should not be conflated with free viewing because the target category imposes strong top-down structure.

The suitability of a dataset for semantic reconstruction depends on the research question. Free viewing may provide richer spontaneous transition structure but weaker control over motivation; visual search provides clearer goals but may cause semantic relations to be dominated by target relevance. A future individual-semantics study should therefore either collect purpose-built data or explicitly model task effects when reusing existing datasets.

### 5.3. Individual Differences in Scene Viewing

Individual differences are not merely noise. de Haas et al. [49] showed stable observer-specific differences in what attracts gaze across complex scenes and found that these differences varied along semantic dimensions; scanpath-network approaches likewise show that participant-level relational patterns can be quantified in reading [50]. These results motivate observer-specific analysis, but they do not demonstrate that a complete individual semantic space can be reconstructed from scanpaths or that gaze-derived structure has already been validated as an individual’s semantic representation. The defensible target is an observer-specific gaze-derived relational structure whose reliability and external semantic validity must be established before it is interpreted as individual semantic alignment.

## 6. A Proposed Framework for Gaze-Derived Relational Geometry

### 6.1. From Raw Fixations to Object-Level Scanpaths

For an image containing identifiable objects, each object can be defined as an area of interest (AOI) with a label and spatial extent. Fixations can then be assigned to AOIs, producing an object-level sequence such as dog →cat → sofa → television. Consecutive fixations within the same AOI may be retained when dwell dynamics are of interest or collapsed when the focus is transitions between distinct objects. Crucially, this mapping is an analytical decision rather than a neutral conversion: tracker quality, event detection, AOI boundaries, overlap rules, and missing samples can all alter the resulting sequence [51,52,53,54].

The first analytical product should be a transition table, not a semantic space. For each transition at time t, the observed destination is recorded together with all plausible candidate destinations and their properties. This candidate-based formulation is essential because it asks why object j was selected instead of the other objects that were available.

### 6.2. Measurement Quality and Preprocessing Before Semantic Inference

A methodological framework must make the measurement chain explicit. Calibration and validation quality should be quantified using accuracy, precision, and data-loss measures rather than assumed from successful tracker setup [51,52,53,54]. Fixation and saccade events should be derived with a stated algorithm and thresholds, and analyses should report how blinks, track loss, off-screen samples, and short or ambiguous events are handled. AOI definitions should be specified before semantic analysis where possible, with reproducible rules for boundaries and overlapping AOIs; sensitivity to alternative AOI padding or assignment rules should be checked when localization uncertainty is non-negligible.

Repeated within-object fixations should be handled according to the inferential target: retaining them preserves dwell/reinspection dynamics, whereas collapsing them yields a transition graph over distinct objects. For reading, the corresponding mapping problem occurs between human regions of interest (usually words) and LM subword tokens. Summing token surprisals is common, but tokenization can change psychometric estimates; analyses should therefore define the word/region aggregation rule and consider tokenization-invariant or marginalized estimates when feasible [27,28]. Table 2 lists preprocessing decisions that should be reported and, for confirmatory analyses, subjected to prespecified sensitivity checks.

### 6.3. Competing Semantic Relation Matrices

For each object pair *i* and *j*, the framework should estimate not one undifferentiated semantic distance but a set of relation-specific dissimilarities *D^(r)^(i, j)*, where *r* indexes a theoretically defined relation family. Depending on the study, these may include taxonomic similarity, thematic/functional association, contextual compatibility, or a model-derived embedding relation. Predictability or task relevance should be retained as transition-level predictors rather than silently folded into the same construct. For embedding-based relations, cosine dissimilarity can be used as one operationalization, provided the embedding source, layer/prompt, pooling, and normalization are specified.

$$D_{cos}^{\left(r\right)}(i,\;j)=1-\frac{{e_{i}^{\left(r\right)}}^{T}\;e_{j}^{\left(r\right)}}{\|e_{i}^{\left(r\right)}{\|}_{2}\;\|e_{j}^{\left(r\right)}{\|}_{2}}.$$

Here $e_{i}^{\left(r\right)}$ and $e_{j}^{\left(r\right)}$ denote the embedding vectors for objects i and j under relation representation *r*, and ‖·${\|}_{2}$ denotes the Euclidean norm. For unit-normalized embeddings, the expression reduces to 1 − $e_{i}^{\left(r\right)}$· $e_{j}^{\left(r\right)}$. We use ‘cosine dissimilarity’ deliberately because 1-cosine similarity is a useful dissimilarity function but is not a metric in the strict mathematical sense in all settings. Competing relation matrices should be entered or compared separately. Agreement across taxonomic, thematic, contextual, and model-derived representations strengthens a broad relatedness interpretation; divergence is scientifically informative because it identifies which relation type is most consistent with gaze. This preserves the construct distinctions developed in Section 2 rather than collapsing them into a single *D_sem_* variable.

### 6.4. Candidate-Based Transition Choice and Critical Confounds

The central methodological error to avoid is equating a high transition probability with semantic relatedness. A transition from object *i* to candidate *j* is influenced by relation-specific semantic variables but also by Euclidean distance between AOIs, saccade direction/amplitude, target eccentricity, AOI area, low-level salience, object recognizability, scene centrality, previous fixations, inhibition of return, and task relevance. Scene-level co-occurrence can additionally make semantically associated objects physically adjacent, creating a structural confound. Regression adjustment reduces measured confounding but does not by itself establish that a semantic–spatial correlation has been causally separated.

The next fixation is fundamentally a discrete choice among the objects simultaneously available at transition *t*. Let $C_{pt}$ denote the candidate set for participant p at transition *t*, let $i_{pt}$ be the currently fixated object, and let $\eta_{ptj}$ be the utility of candidate *j*. A conditional-logit/multinomial-softmax formulation normalizes the probability over the full candidate set [55,56]. A hierarchical extension can include participant-specific slopes for semantic, spatial, or salience effects and image/object structure when these terms vary meaningfully across alternatives or contexts. Random intercepts that are constant for every alternative within the same choice set cancel under softmax normalization and should not be added mechanically.

$$\eta_{ptj}=\sum_{r=1}^{R}\beta_{r}\;D^{\left(r\right)}(i_{pt},\;j)+\beta_{s}\;D_{spatial}(i_{pt},\;j)+{\beta_{z}}^{T}\;z_{ptj}+{b_{p}}^{T}\;x_{ptj}+{c_{I_{pt}}}^{T}\;x_{ptj}.$$

$$P(J_{pt}=j\;|\;C_{pt})=\frac{exp(\eta_{ptj})}{\sum_{k\;\in \;C_{pt}}\;exp(\eta_{ptk})},\;j\;\in \;C_{pt}.$$

Here $\beta_{r}$ values are fixed effects for the *R* relation-specific dissimilarity predictors, $\beta_{s}$ is the fixed spatial-distance effect, and $\beta_{z}$ is the fixed-effect vector for candidate-specific visual and history covariates $z_{ptj}$ (for example AOI size, salience, eccentricity, and prior fixation). The vectors $b_{p}$ and $c_{I_{pt}}$ contain participant- and image-specific random slopes, respectively, for the candidate-varying predictor subset $x_{ptj}$; trial-level random slopes can be added analogously when the sampling design supports them. A random intercept shared by every candidate in a single choice set would cancel under the softmax normalization and is therefore not separately identifiable. Evidence for a semantic contribution requires the relevant relation-specific coefficient to improve prediction or likelihood after these controls and to generalize under held-out evaluation. However, when semantic and spatial structure are strongly collinear in natural scenes, coefficient adjustment remains observational. Stronger identification requires experimental designs that counterbalance object locations or orthogonally manipulate semantic relatedness and spatial distance across otherwise comparable scenes (Figure 1).

### 6.5. Residualized Gaze Relations

A second route is to construct an observed transition matrix and remove expected transition structure attributable to prespecified nonsemantic variables. The residual association between object pairs can then be treated as a candidate gaze-derived relational matrix. ‘Residual’ is crucial: the raw transition matrix is behavioral, and the unexplained component after explicit controls is at most an estimate of relational structure. Residualization does not solve unmeasured or structurally inseparable confounding; its interpretation is strongest when supported by counterbalanced stimuli, out-of-sample prediction, and independent semantic validation.

This residual matrix may be symmetrized when the theoretical focus is undirected semantic relatedness, or retained as directed when asymmetric associations are meaningful. Directionality may be cognitively informative: cup → coffee machine and coffee machine → cup need not have equal probabilities, even if a standard cosine similarity matrix is symmetric.

### 6.6. Representational Similarity Analysis

Representational similarity analysis (RSA) offers a natural bridge between gaze and model spaces [57,58]. For a fixed object set one can construct (a) one or more model relation/dissimilarity matrices, (b) a gaze-derived relational-distance matrix, and (c) nuisance matrices such as spatial distance. Matrix entries are not independent observations, so inferential procedures should preserve the dependency structure rather than apply ordinary tests to vectorized cells. Appropriate options include permutation of object/stimulus labels (or restricted permutations respecting the experimental unit), regression RSA with permutation-based inference, and cross-validated model comparison. Spatial nuisance structure can be included in regression RSA or residualized using training data within each cross-validation fold to avoid leakage.

At the participant level, a separate gaze-derived matrix can be estimated for each observer, but an alignment score should be interpreted only after reliability is demonstrated. Split-half or test–retest reliability should be reported with uncertainty, and bootstrap or hierarchical intervals can quantify estimation error. A noise ceiling can indicate the maximum model-observer correspondence permitted by the reliability of the behavioral matrix [57,58,59,60]. Sparse matrices may benefit from hierarchical shrinkage/partial pooling, provided individual estimates are not forced toward the group so strongly that genuine heterogeneity is erased. All participant-level model comparisons should be evaluated on held-out trials/images or otherwise protected from circular hyperparameter selection.

### 6.7. Learning Embeddings from Gaze Sequences

Dimensionality reduction or graph-embedding methods can be applied after a relational matrix has been estimated. Multidimensional scaling is particularly interpretable because it starts from pairwise distances. Spectral methods or UMAP can aid visualization, although visual topology should not be confused with validated psychological dimensions. Sequence-learning approaches inspired by Word2Vec are also possible if object fixations are treated analogously to tokens in behavioral contexts, but such use requires a clear definition of context window, repeated exposure, transition direction, and sampling balance. GloVe-like methods likewise require an explicit co-occurrence matrix and weighting scheme. These methods should be presented as candidate computational tools rather than as established gaze-to-semantics pipelines.

### 6.8. Required Independent Validation

Independent validation is a requirement for any claim that gaze-derived relational structure reflects semantic organization. A matrix estimated from gaze becomes semantically interpretable only if it predicts data not used to construct or tune it. Suitable validation measures include free association, pairwise relatedness judgments, semantic sorting, semantic differential ratings, category judgments, or task-specific conceptual similarity measures (Table 3). For observer-specific claims, validation must also be observer-specific wherever feasible; a group-level correlation cannot establish recovery of an individual’s semantic organization [61].

The inferential target is therefore a triangulation of three independently obtained structures: model-derived semantic relations, gaze-derived relational structure, and independently measured human semantic structure. Convergence between gaze and independent human measures validates the behavioral estimate; only after this step should correspondence with model geometry be interpreted as human–model semantic alignment. Figure 2 summarizes the framework and emphasizes that the human behavioral and model representational pathways remain distinct until the comparison stage.

## 7. Semantic Scanpaths and Vision–Language Models

Recent work makes semantic analysis of scanpaths more concrete. Kerkouri et al. [40] proposed a semantic scanpath similarity framework in which fixation regions are described using vision–language models and scanpaths are compared using embedding-based and lexical NLP metrics. Their results suggest that semantic similarity captures variance partly independent of purely geometric scanpath alignment. Related gaze-informed NLP work shows that scanpaths themselves can be generated, modeled, or used as task signals [39,43,44]. These approaches demonstrate that modern models can attach interpretable semantic content to attended regions or sequences, but they do not by themselves reconstruct an observer’s semantic memory.

This development is particularly relevant to labeled-image paradigms. When object labels are known, semantic representations can be computed directly from labels; when labels are uncertain or the attended region contains relational information that a category name misses, a vision–language description may provide a richer representation. A hybrid analysis could compare predefined object labels with VLM-derived local descriptions to test whether gaze is better explained by categorical identity, thematic scene context, or visually grounded semantics.

## 8. Human–LLM Semantic Alignment and Observer-Specific Relational Differences

### 8.1. Population Semantics Versus Observer-Specific Relational Structure

Corpus-trained language models learn statistical structure aggregated across many texts and speakers. Their semantic geometry is therefore population-derived and representation-dependent. Human semantic knowledge is also shaped by shared language and culture, but individual experience, expertise, goals, and personal associations can produce systematic deviations from population averages. Stable observer-specific variation in gaze allocation [49] and participant-level scanpath networks [50] motivates testing whether some of these deviations are reflected in reliable gaze-derived relational structure; it does not establish that scanpaths are themselves individual semantic representations.

Accordingly, we replace the stronger notion of an ‘individual semantic gap’ with observer-specific semantic alignment or gaze-derived relational divergence. A mismatch between a participant’s validated behavioral relational structure and a model’s geometry does not imply that either representation is wrong. It may reflect experience, task interpretation, relation type, context, or model limitations. The scientific goal is to quantify such mismatches and test when they predict independent judgments, communication, or task performance.

### 8.2. Quantifying Alignment

For participant *p*, let *D_gaze_,p* be a reliable and independently validated gaze-derived relational-distance matrix and let *D_model_^(r)^* denote a corresponding model matrix for relation family *r*. An alignment coefficient can be estimated by comparing held-out or cross-validated matrix structure, with nuisance variables handled inside the inferential procedure. Because pairwise cells are dependent, uncertainty should be obtained with label-/stimulus-level permutation, bootstrap over independent experimental units, or hierarchical modeling rather than treating all matrix cells as independent. Differences in alignment across participants, semantic relation models, or tasks can then be tested with hierarchical statistics, accompanied by reliability estimates and a behavioral noise ceiling.

This formulation separates measurement from interpretation. The eye tracker provides fixation sequences; the statistical model provides a gaze-derived relational structure; the language model provides a semantic geometry; and the alignment statistic compares them. No single step is itself “the individual semantic space.” The interpretation emerges only when the gaze-derived structure is reliable and externally validated.

### 8.3. Potential Applications

If validated, individual human–model alignment measures could support several research applications. They could identify semantic domains in which model representations systematically diverge from a user’s associations, examine how expertise reshapes conceptual organization, or test whether personalized interaction reduces misunderstandings. In human–AI interaction, the value would lie less in making an LLM imitate every idiosyncrasy than in detecting when apparently shared words occupy different relational neighborhoods for the model and the user.

Such applications also raise ethical and privacy concerns. Gaze data can reveal sensitive patterns of attention and should not be treated as innocuous telemetry. Any attempt to infer individual semantic structure requires informed consent, transparent explanation of the inference, strict data minimization, and caution about overinterpreting noisy person-specific estimates.

## 9. Methodological Challenges and Interpretation Limits

### 9.1. Processing Measures Are Not Representations

The most fundamental limitation is the processing–representation distinction. Embeddings and hidden states are representational objects; eye movements are behavioral events generated during online processing. A representation can influence processing without mapping one-to-one onto fixation duration or scanpath structure. Conversely, failure to predict gaze does not prove that a representation lacks semantic information. Cognitive evaluation should therefore be interpreted as evidence about behavioral relevance under a particular task, not as a complete assay of semantic content.

### 9.2. Cross-Linguistic and Cross-Participant Variability

Cross-linguistic corpora demonstrate that reading behavior varies with orthography, morphology, word segmentation, and language-specific reading strategies. MECO provides an important platform for testing whether model–gaze relationships generalize across languages [32]. Recent multilingual scaling analyses likewise show that apparent model–human alignment can differ across early and late eye-movement measures [26]. Individual differences create an additional level of variation. Aggregating participants may improve signal-to-noise for population-level evaluation but can erase exactly the structure needed for observer-specific analyses. Future work should report population effects, participant-level reliability, and uncertainty rather than presenting a single correlation as a stable individual characteristic.

### 9.3. Dataset Scale and Sampling

Observer-specific relational estimation is data-hungry. A participant must encounter enough objects and object pairs across enough contexts to estimate a stable matrix. Unequal object frequencies and sparse transitions can create misleading geometry. Experimental designs should therefore balance semantic categories, spatial arrangements, and transition opportunities, or use hierarchical regularization that shares information without collapsing individual differences. Sample-size planning should be driven by reliability of the participant-level matrices and predictive performance on held-out trials, not only by the number of recorded fixations.

### 9.4. Object Identification and Semantic Granularity

Scene-based approaches depend on deciding what the units of meaning are. Object detectors provide convenient category labels, but labels such as ‘person,’ ‘cup,’ or ‘chair’ discard attributes, roles, relations, and event context. The relevant semantic unit may be ‘woman holding cup,’ ‘coffee cup,’ or ‘object used for drinking,’ depending on the task. Vision–language descriptions can enrich granularity but introduce model-generated interpretation into what is supposed to be a human behavioral measure. AOI boundaries and overlap rules introduce an additional measurement layer [53]. Analyses should therefore distinguish annotations supplied independently of gaze from descriptions generated by the model being evaluated and should test whether semantic conclusions survive plausible alternative AOI definitions.

### 9.5. Statistical Circularity

Circularity is a serious risk when the same model is used both to define the semantic labels of fixated regions and to provide the semantic geometry against which gaze is evaluated. A strong design should separate annotation and evaluation models, use human-validated labels, or test robustness across multiple representation sources. Likewise, hyperparameters should not be chosen solely to maximize model–gaze correspondence on the same data used for the final inference.

## 10. A Research Agenda for LLM–Eye-Tracking Studies

The intersection of large language models and eye tracking is moving from simple correlation toward richer questions about representational alignment. Five priorities appear especially important.

Disentangle semantic relatedness from predictability: Reading studies should include surprisal, lexical frequency, length, and context-sensitive semantic predictors in the same statistical framework rather than interpreting isolated correlations.

Model transitions as candidate-normalized choices: In scenes, each next fixation should be modeled relative to the simultaneously available candidate objects using conditional/multinomial choice probabilities. Spatial, visual, history, and task variables should be explicit predictors, with participant heterogeneity modeled through identifiable hierarchical effects.

Measure reliability and uncertainty before individualization: Participant-specific relational matrices and alignment scores should be interpreted only when split-half or test–retest reliability is sufficient, uncertainty is quantified, and model comparison respects matrix dependence through permutation, cross-validation, and, where useful, noise ceilings or hierarchical shrinkage.

Require independent validation of gaze-derived geometry: Free association, sorting, semantic judgments, or domain-specific conceptual measures are necessary to establish that a gaze-derived residual/relational structure reflects semantic organization rather than another stable attentional trait. Observer-specific semantic claims should not be made without this external validation.

Compare multiple semantic relation models: Static embeddings, contextual encoders, modern embedding models, taxonomic resources, thematic/associative measures, and vision–language representations can encode different relations. Human–model alignment should therefore be reported separately by relation family and representation rather than summarized as one generic semantic-distance effect.

Design semantic–spatial dissociations experimentally: When feasible, counterbalance object locations or orthogonally manipulate semantic relatedness and spatial distance so that a semantic effect is not identified solely by regression adjustment in naturally correlated scenes.

A further priority is the development of preregistered benchmarks in which the same labeled scenes, gaze-processing pipeline, nuisance covariates, semantic models, and validation tasks are shared across laboratories. Such standardization would make it possible to distinguish robust effects from choices that are specific to one dataset or preprocessing pipeline.

## 11. Conclusions

Eye tracking provides a valuable cognitive benchmark for language-model semantics because it connects computational representations with behavior produced during real-time language and scene processing. The literature shows that model-derived semantic relatedness and surprisal can both account for eye-movement variance, while cognitive-prediction and gaze-informed NLP frameworks demonstrate that linguistic representations differ in their behavioral alignment. Recent work also shows that this alignment is contingent on model scale, layer, tokenization, task, and eye-movement measure [23,24,25,26,27,28,35,37,39,44,62]. These findings justify cognitive evaluation, but they do not make eye movements a direct or definitive measure of semantic representation. Recent gaze-focused NLP work further extends this agenda by using reading-related gaze representations to align language models with human behavior and by modeling how contextual linguistic information contributes to the selection of subsequent fixation targets [63,64].

The extension from reading to labeled visual scenes remains promising, but the unit of analysis should be a candidate-based transition rather than an isolated binary outcome. Successive fixations can be transformed into object-level relational data and compared with multiple, explicitly defined semantic-relation models. Spatial proximity, salience, object size, scene structure, gaze history, task goals, preprocessing, and candidate availability must be modeled, and semantic–spatial relations should be experimentally dissociated whenever possible. Candidate-normalized choice models and RSA can then quantify relational correspondence, provided matrix dependence, reliability, uncertainty, and out-of-sample generalization are respected.

The resulting framework offers a principled way to study observer-specific human–model semantic alignment without assuming that scanpaths are semantic representations. The strongest inferential chain is explicitly three-part: reliable gaze-derived relational structure, independent behavioral validation of its semantic content, and only then comparison with model geometry. If future studies establish this reliability and external validity, gaze-derived relational geometry could complement explicit semantic judgments, free association, and conventional model benchmarks. Its role is therefore as one component of a multimethod science of how computational and human systems organize and process meaning, not as a replacement for intrinsic or extrinsic evaluation.

## Figures and Tables

**Figure 1:**
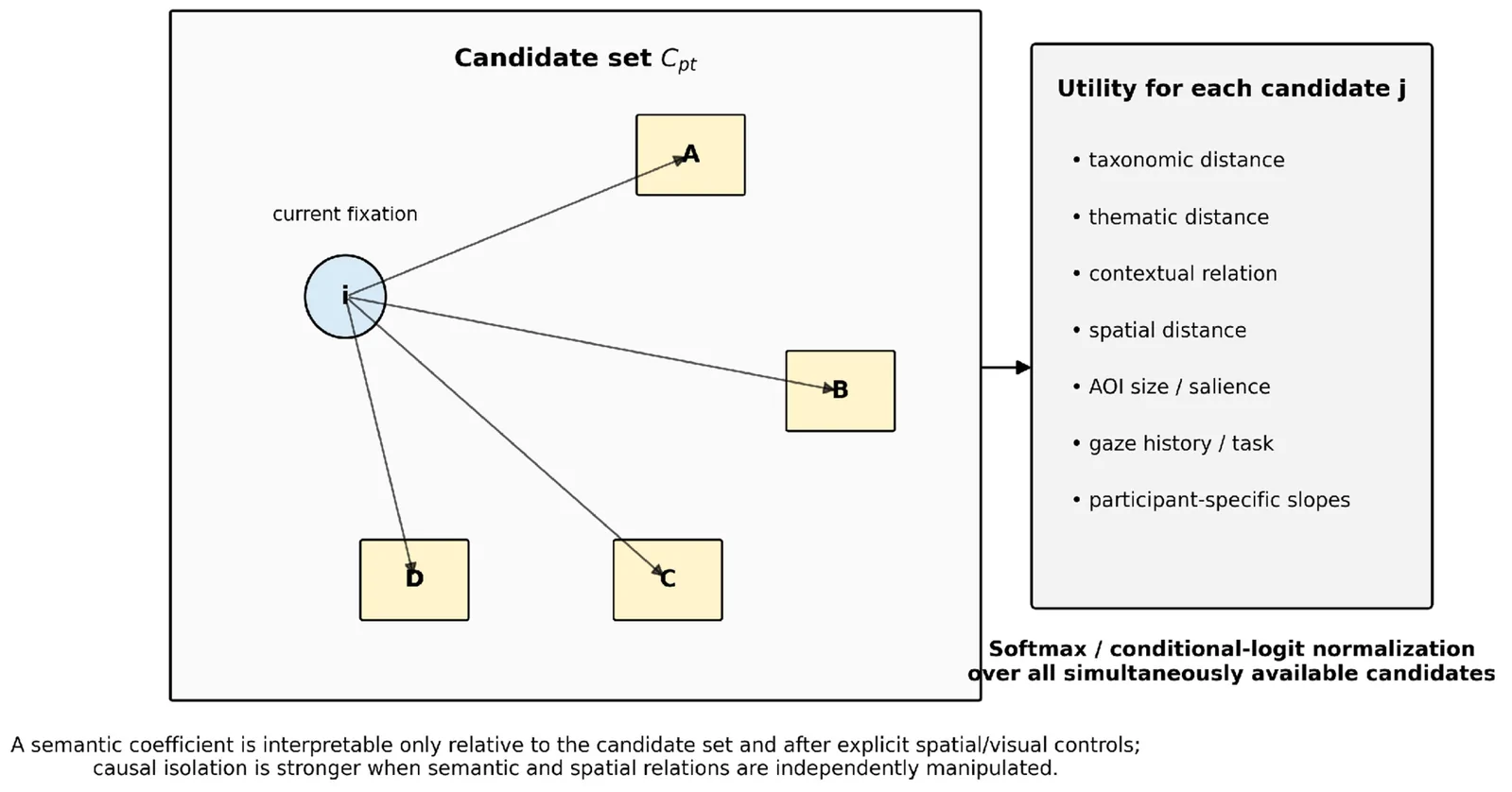
Candidate-normalized next-fixation model. Each transition is modeled as a choice among simultaneously available AOIs, with separate semantic-relation, spatial, visual, history, task, and participant-level effects. Experimental counterbalancing is preferred when semantic and spatial structure are intrinsically correlated.

**Figure 2:**
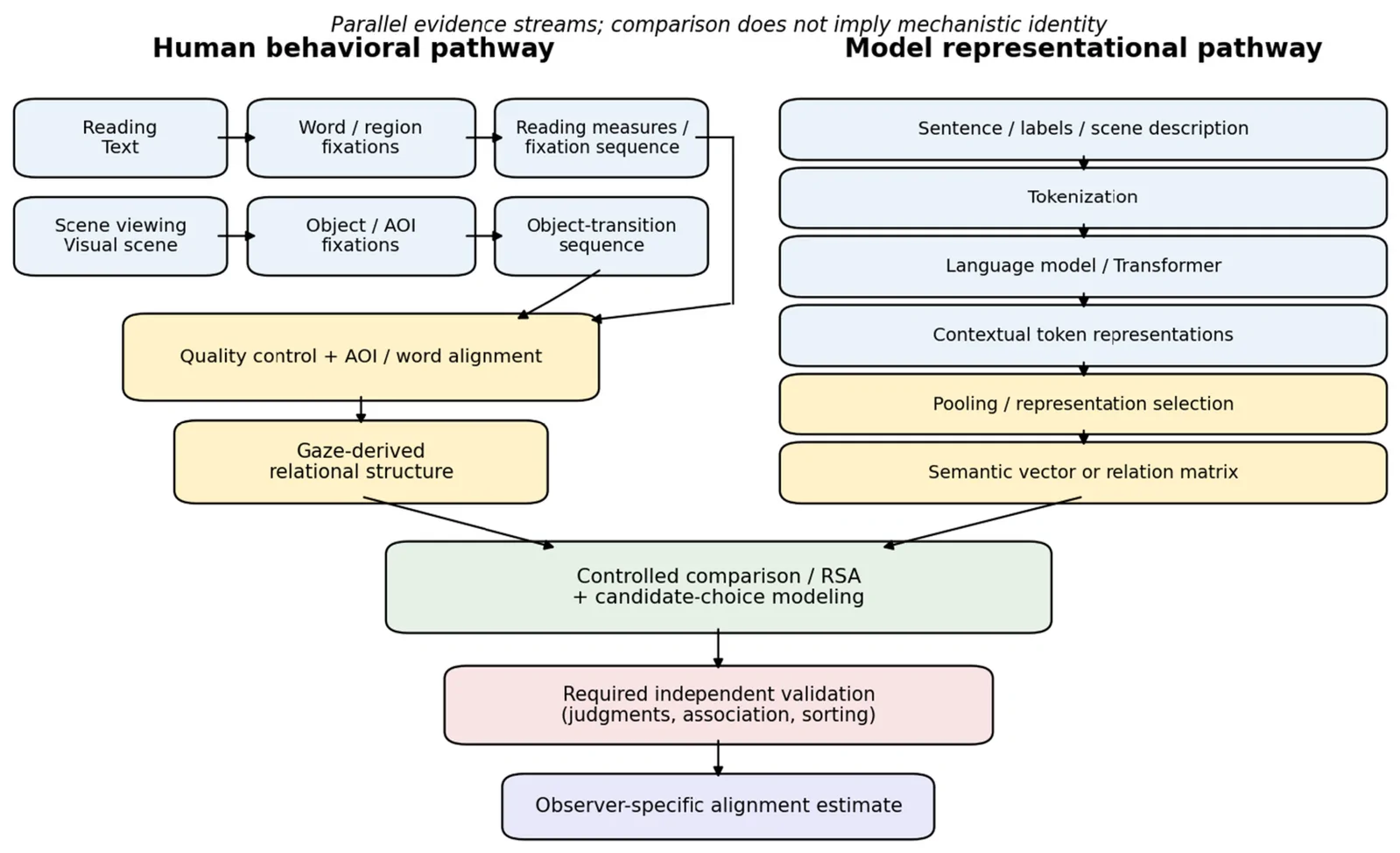
Proposed framework for observer-specific semantic alignment across reading and scene-viewing paradigms. Human fixation sequences and model representations are treated as parallel but non-equivalent evidence streams. Linguistic or scene-derived symbolic input is tokenized and transformed into contextual model representations, from which semantic vectors or relation matrices are derived. Human and model pathways are compared only after task-appropriate preprocessing and control of relevant nuisance variables; independent behavioral validation is required before observer-specific semantic interpretation.

**Table 1:** Representative recent work linking gaze and computational language representations.

Study	Model/Representation	Eye-Tracking Target	Main Comparison	Interpretive Contribution
Bensemann et al. [35]	Pretrained transformers	Reading dwell time	Layer-wise self-attention vs. gaze	Alignment strongest in early layers; not explained by parameter count alone.
Ding et al. [36]	CogBERT	Reading-related cognitive features	Cognition-guided pretraining	Shows that gaze/cognitive information can be incorporated into LM training.
Oh & Schuler [24,25]	GPT-2/GPT-Neo/OPT families	Naturalistic reading times	Surprisal vs. model size/training exposure	Larger/better-perplexity models are not necessarily more human-like psychometrically.
de Varda & Marelli [26]	Transformer LMs, 10 languages	Early and late eye-movement measures	Surprisal vs. parameter scale	Scale effects differ across processing stages.
Sun et al. [23]	Contextual semantic similarity	Reading eye movements	Semantic similarity beyond standard controls	Supports interpretable semantic predictors while preserving construct specificity.
Zhang & Hollenstein [37]	BERT/ALBERT	FFD and total reading time	Eye-tracking attention masks in QA	Gaze information can complement model representations; gains are architecture-sensitive.
Giulianelli et al. [27]	Tokenized LMs	Word/region reading measures	Token-to-region surprisal alignment	Tokenization is a methodological, not merely implementation, issue.
Shubi et al. [39]	Transformer scanpath models	Reading scanpaths	Decoding reading goals	Task goals are recoverable from gaze and must be modeled as substantive variance.
Kerkouri et al. [40]	VLM + NLP metrics	Scene scanpaths	Semantic vs. geometric scanpath similarity	Semantic description captures information partly distinct from spatial alignment.

**Table 2:** Minimum measurement and preprocessing checks for gaze-derived relational analyses.

Stage	Decision to Report	Primary Risk	Recommended Check
Calibration/validation	Accuracy, precision, data loss; validation procedure	AOI misassignment and biased spatial estimates	Report participant/session quality; define exclusion or re-calibration criteria [51,52,53,54].
Event detection	Fixation/saccade algorithm and thresholds	Algorithm-dependent fixation counts/durations	State parameters; test plausible alternative settings when conclusions depend on them.
AOI construction	Source, size, shape, padding, dynamic/static status	Construct contamination; unequal opportunity to be fixated	Define independently of gaze where feasible and document geometry [53].
AOI overlap/boundary cases	Tie-breaking or probabilistic assignment rule	Arbitrary candidate identity	Prespecify rule; sensitivity analysis for ambiguous samples.
Repeated within-AOI fixations	Retain, collapse, or model separately	Mixing dwell dynamics with object transitions	Match preprocessing to the intended behavioral construct.
Missing/off-screen data	Blink, track-loss, and truncation handling	Non-random transition loss	Report missingness by participant/image; test whether loss predicts condition.
Candidate set	Which objects were available at each transition	Biased denominator in choice probabilities	Construct choice set at each time point; exclude unavailable/occluded candidates explicitly.
Reading LM alignment	Word-to-subword aggregation/marginalization	Tokenizer-dependent surprisal and similarity	Define focal region; report aggregation; test alternative alignment when feasible [27,28].
Reliability/sensitivity	Split-half/test–retest; preprocessing perturbations	Unstable participant-level geometry	Do not interpret individual scores below a justified reliability threshold.

**Table 3:** Evaluation sources and what they can establish.

Source	Primary Signal	Individual	Strength	Main Limitation
Word-similarity judgments	Explicit pairwise semantic judgment	Yes	Direct semantic report	Artificial task; limited item coverage
Downstream NLP tasks	Task performance	Usually no	Functional utility	Task-dependent and indirect
Reading eye movements	Online processing difficulty	Yes	Naturalistic, time-resolved	Predictability and lexical confounds
Scene gaze transitions	Attentional selection among objects	Yes	Object-level behavioral structure	Spatial, visual, and task confounds
Free association/sorting	Behavioral semantic organization	Yes	Useful validation target	Explicit task demands
Proposed residualized gaze geometry	Controlled object-to-object relational structure	Yes	Potential implicit individual measure	Requires reliability and independent validation

## Data Availability

No new data were created or analyzed in this study. Data sharing is not applicable to this article.
